# Supplementary figures and images for: Structural analysis of urinary light chains and proteomic analysis of hyaline tubular casts in light chain associated kidney disorders
Source: PeerJ. 2019 Oct 2;7:e7819. doi: 10.7717/peerj.7819 (PMC6778432; doi:10.7717/peerj.7819)

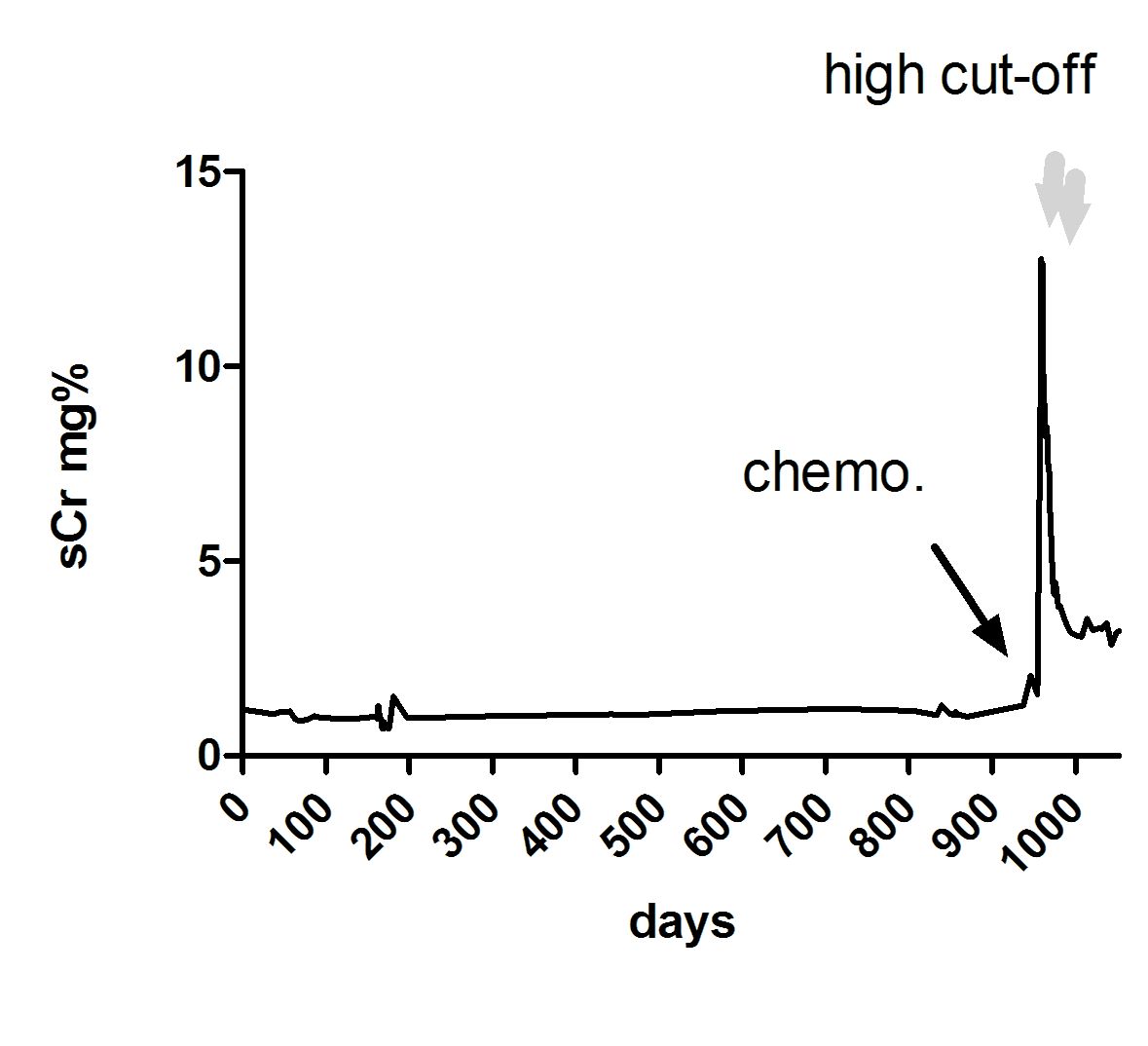

Supplement: Figure S1 — Time point of cast harvesting for proteome analysis was shortly before initiation of dialysis. Two dialysis sessions with high cut-off membranes are depicted by grey arrows. [file peerj-07-7819-s001.jpg]
